# Indications for Emergency Intervention, Mode of Delivery, and the Childbirth Experience

**DOI:** 10.1371/journal.pone.0169132

**Published:** 2017-01-03

**Authors:** Jonathan E. Handelzalts, Avigail Waldman Peyser, Haim Krissi, Sigal Levy, Arnon Wiznitzer, Yoav Peled

**Affiliations:** 1 School of Behavioral Sciences, The Academic College of Tel Aviv-Yaffo, Tel Aviv, Israel; 2 The Helen Schneider Hospital for Women, Rabin Medical Center, Petach Tikva, Israel; 3 Sackler Faculty of Medicine, Tel Aviv University, Tel Aviv, Israel; Harvard Medical School, UNITED STATES

## Abstract

**Background:**

Although the impact of emergency procedures on the childbirth experience has been studied extensively, a possible association of childbirth experience with indications for emergency interventions has not been reported.

**Objectives:**

To compare the impacts on childbirth experience of ‘planned’ delivery (elective cesarean section and vaginal delivery) versus ‘unplanned’ delivery (vacuum extraction or emergency cesarean section); the intervention itself (vacuum extraction versus emergency cesarean section); and indications for intervention (arrest of labor versus risk to the mother or fetus).

**Study design:**

A total of 469 women, up to 72 hours post-partum, in the maternity ward of one tertiary health care institute completed the Subjective Childbirth Experience Questionnaire (score: 0–4, a higher score indicated a more negative experience) and a Personal Information Questionnaire. Intra-partum information was retrieved from the medical records. One-way analysis of variance and two-way analysis of variance, followed by analysis of covariance, to test the unique contribution of variables, were used to examine differences between groups in outcome. Tukey's Post-Hoc analysis was used when appropriate.

**Results:**

Planned delivery, either vaginal or elective cesarean section, was associated with a more positive experience than unplanned delivery, either vacuum or emergency cesarean section (mean respective Subjective Childbirth Experience scores: 1.58 and 1.49 vs. 2.02 and 2.07, *P* <0.01). The difference in mean Subjective Childbirth Experience scores following elective cesarean section and vaginal delivery was not significant; nor was the difference following vacuum extraction and emergency cesarean section. Interventions due to immediate risk to mother or fetus resulted in a more positive birth experience than interventions due to arrest of labor (Subjective Childbirth Experience: 1.9 vs. 2.2, *P* <0.01).

**Conclusions:**

Compared to planned interventions, unplanned interventions were shown to be associated with a more negative maternal childbirth experience. However, the indication for unplanned intervention appears to have a greater effect than the nature of the intervention on the birth experience. Women who underwent emergency interventions due to delay of birth (arrest of labor) perceived their birth experience more negatively than those who underwent interventions due to risk for the mother or fetus, regardless of the nature of the intervention (vacuum or emergency cesarean section). The results indicate the importance of follow-up after unexpected emergency interventions, especially following arrest of labor, as negative birth experience may have repercussions in a woman's psychosocial life and well-being.

## Introduction

Childbirth is an important life event for women. A positive delivery experience may have a long lasting effect on a woman's feelings of self-worth [1]. A negative delivery experience can be disempowering and lead to maternal distress or posttraumatic stress disorder (PTSD) and postpartum depression [2–5]. A recent meta-analysis demonstrated that the risk factors most strongly associated with post-partum PTSD were negative subjective birth experience, operative birth [assisted vaginal or cesarean section (CS)], lack of support, and dissociation [6].

Common interventions during delivery are vacuum extraction (VE), forceps delivery, and emergency CS performed when VE/forceps delivery is not feasible or failed. The performance of fewer interventions in delivery was reported to be associated with a more positive childbirth experience [7, 8]. The performance of more obstetric interventions was found to be associated with a traumatic experience [9]. However, other studies have suggested that the degree of women's involvement in decision-making, support during labor, effective analgesia [10] and personal expectations [11] may have greater impact than mode of delivery on childbirth experience.

Psychological variables associated with the childbirth experience, such as involvement in decision-making and personal expectations, may be associated with the indication for intervention, as well as with the type of intervention. Thus, although the impact of emergency procedures on the childbirth experience has been studied extensively, only one study discussed the psychological aspect of interventional indications. However, the subject of that study was not the childbirth experience, but rather expectations and predictions regarding childbirth [12]. Indications for operative delivery can be classified as indications associated with risk to the mother or the fetus and indications associated with arrest of labor. The distress associated with operative delivery may be intensified in interventions associated with arrest of labor, and lead to a more negative childbirth experience. For such deliveries, a sense that something could have been done differently may cause women to feel responsible for the intervention. In contrast, for emergency procedures resulting from indications due to a threat to the mother or to the baby, a woman may feel that the intervention was not associated with her own actions.

In the present study, we hypothesized that perceptions of childbirth experience following ‘planned’ delivery [elective CS and vaginal delivery (VD)] will be more positive following ‘unplanned’ delivery [vacuum extraction (VE) or emergency cesarean section]. Further, we hypothesized that childbirth experience will differ according to the intervention itself (VE will result in better childbirth experience than emergency CS), and according to indications for intervention (risk to the mother or fetus will result in better childbirth experience than arrest of labor).

## Materials and Methods

### Participants and procedure

This cross-sectional questionnaire study was conducted from September 2014 to June 2015 at a tertiary health care institute in Israel. A convenience sample was established of women hospitalized in the maternity ward (up to 72 hours post-partum) who agreed to participate in the study. For power considerations, we sampled equal sized groups, despite the different prevalences of mode of birth in the population. We aimed to recruit at least 80 participants for each group. In practice, we allowed for larger samples when possible.

Women who agreed to participate received a general explanation regarding the study, and signed an informed consent form. Exclusion criteria were preterm delivery before 37 weeks, pregnancy complications (intrauterine growth retardation, diabetes, and pregnancy-induced hypertension/preeclampsia) before commencing active labor, unfavorable delivery outcomes (such as Apgar scores of 7 and below, neonatal abnormalities and the need for neonatal intensive care observation), multiple pregnancy and emergency CS after failed VE.

The study was approved by the Rabin Medical Center institutional review board (0126–14 -RMC).

Of 533 women who were approached to participate, 27 (5%) declined. In addition, 37 participants (6.9%) who answered less than 70% of the questionnaires were excluded from the analysis. The remaining 469 women comprised the study group. Ages ranged from 19 to 47 years (mean 31.0±5.0).

"Planned" birth procedures were defined as: VD (n = 132) and elective CS (n = 103). "Unplanned" procedures were defined as emergency CS (n = 120) and VE (n = 114). "Unplanned" procedures were defined as procedures preformed after the commencing of active labor.

### Descriptions of type of indications

Risk to mother or fetus: This included non-reassuring fetal heart monitor, cord prolapse, non-vertex presentation in delivery, placenta previa in delivery, acute severe preeclampsia (after commencing of active labor), and placenta abruption.

Arrest of labor: This included arrest of descent, arrest of dilatation, prolonged second stage, and maternal exhaustion.

**Fig 1:**
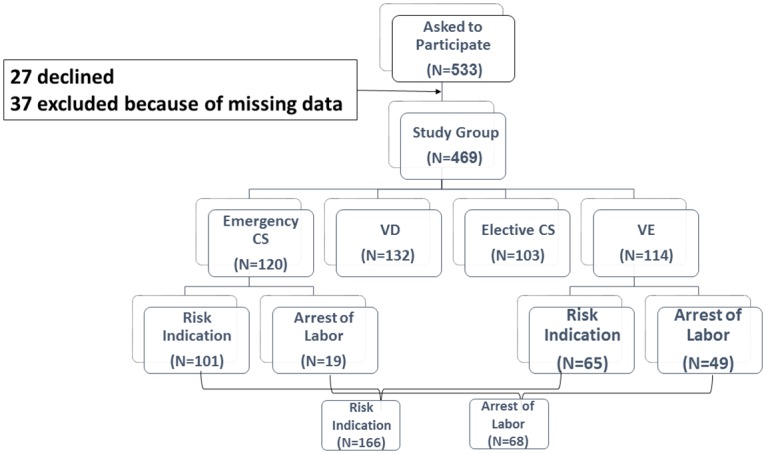
Participant flow diagram.

The distribution of the study groups is presented in Fig 1.

### Measures

Personal Information Questionnaire: A questionnaire that included data on age, education, country of origin, family status, gestational age at delivery, and number of prior births.

Subjective Childbirth Experience Questionnaire (SCE): A questionnaire that assesses the subjective experience of childbirth [13]. The SCE consists of 10 items that rate perception of the childbirth event, and that are rated on a 5-point scale, ranging from not at all (0) to high severity (4). The score is based on the mean response of the 10 items (0–4), with a high score denoting a negative experience. The questionnaire is stress-oriented, and all its items refer to the negative quality of the childbirth experience. A sample question was: "To what extent did you feel helplessness during your birth?" In the current study, the internal consistency was 0.82.

Intra-partum information was obtained from the medical records of the participants, and included the mode of delivery [VD, VE, emergency or elective CS] and indications for VE or CS.

### Statistical analysis

Continuous and categorical demographic characteristics were compared between study groups using a one-way ANOVA and a Chi-square test, respectively. Pearson correlation coefficients were calculated between outcome and demographic variables. One-way ANOVA and two-way ANOVA, followed by analysis of covariance, to test the unique contribution of variables, were used to examine differences between groups in outcome. Tukey's Post-Hoc analysis was used when appropriate. Power analyses showed that groups of size 80 (assuming equal size for maximum power) would detect a medium effect size with a probability of at least 85% for all statistical analyses performed. Data were analyzed using the statistical software package SPSS 21.0 (SPSS Inc., Chicago, IL).

## Results

Background information on the study participants, according to mode of delivery, is presented in Table 1. Differences according to mode of delivery were observed in women's age, education, gestational age and parity. These variables were therefore held as covariates in further analyses.

**Table 1:** Background characteristics of study participants by mode of delivery.

Characteristic	Mode of delivery					
	Vaginal (n = 132)	Vacuum (n = 114)	Emergency CS (n = 120)	Elective CS (n = 103)	F/ χ2	*P*
Women's age (yrs.)	30.3 (5.1)^a^^,^^b^	29.1 (4.8)^a^	31.0 (4.7)^b^^,^^c^	33.6 (4.5)^c^	15.25	<.001
Education (yrs.)	15.0 (2.4)^a^^,^^b^	14.3 (2.3)^a^	14.6 (2.5)^a^^,^^b^	15.2 (2.2)^b^	2.8	.039
Gestational age (wks.)	39.0 (1.6)^a^	39.1 (1.8)^a^	37.8 (2.7)^b^	38.4 (0.8)^b^	14.5	<.001
Parity	2.6 (1.4)^a^	1.5 (1.0)^b^	2.2 (1.7) ^a^	2.7 (1.4)^b^	13.9	<.001
Marital status					4.1	.250
Married	108 (96%)	98 (96%)	107 (93%)	90 (90%)		
Not married	5 (4%)	4 (4%)	8 (7%)	10 (10%)		

All values are mean (SD) or n(%).

^a,b,c^ Groups with common indices did not differ significantly by Tukey's test for multiple comparisons.

### Mode of delivery and birth experience

Women who underwent "planned" delivery: VD (SCE, mean±SD: 1.58±0.73) or elective CS (1.49±0.76) reported a significantly more positive childbirth experience than women who underwent "unplanned" delivery: VE (2.02±0.67) or emergency CS (2.07±0.85) (F (3, 465) = 18.2, *P* <0.01, η^2^ = 11.7%). No statistically significant differences were noted in birth experience between women who underwent VD and elective CS or between women who underwent VE and emergency CS. Similar results were obtained when controlling for age, education, gestational age and parity.

### Indication for intervention in "unplanned" delivery, and birth experience

For the women who underwent unplanned procedures (N = 234), we analyzed SCE scores according to the indication that led to the medical intervention (risk to mother or fetus, or arrest of labor) and the mode of delivery (VE or emergency CS). The results are presented in Table 2. Regarding indications, childbirth experience was found to be more negative for women for whom the indication for "unplanned" delivery was arrested labor than for women for whom the indication was risk to the mother or fetus. Similar results were obtained when controlling for age, education, gestational age and parity.

**Table 2:** Subjective Childbirth Experience Questionnaire scores according to mode of "unplanned" delivery and indication of intervention.

Indication of intervention	Mode of "unplanned" delivery		
	Emergency Cesarean Section	Vacuum Extraction	Total
**Arrest of labor**	2.4 (0.6) (n = 19)	2.2 (0.6) (n = 49)	2.2 (0.6) (n = 68)
**Risk indication**	2.0 (0.9) (n = 101)	1.9 (0.7) (n = 65)	1.9 (0.8) (n = 166)
**Total**	2.1 (0.8) (n = 120)	2.0 (0.7) (n = 114)	2.0 (0.8) (n = 234)

Values are presented as mean (SD). Mode of "unplanned delivery: F (1,230) = 2.207, p = NS. Indication of intervention: F (1,230) = 9.576, *P* <0.01, η^2^ = 4.3%. Mode by indication interaction: F (1,230) = 0.825, p = NS.

Examination of the responses to the 10 items that comprised the SCE Questionnaire show higher scores (more negative perception) among women who underwent interventions due to arrested labor than due to maternal or fetal risk, for the following: difficulty of birth, less control, and feeling of helplessness (Table 3). No difference in other SCE items was found.

**Table 3:** Mean responses to SCE Questions according to indication for "unplanned" delivery.

SCE Questions	Arrest		Risk		*F*	*P*
	M	SD	M	SD		
To what extent did you feel that the delivery was difficult?	**3.5**	**.7**	**2.9**	**1.2**	**18.1**	**<.001**
To what extent did you feel that you were in control?+	**2.0**	**1.1**	**1.5**	**1.3**	**10.4**	**.001**
To what extent did you feel helpless during delivery?	**3.0**	**1.1**	**2.4**	**1.4**	**8.5**	**.004**
To what extent did you feel terror during delivery?	**2.3**	**1.4**	**2.1**	**1.5**	**1.1**	**.300**
To what extent did you feel fear during delivery?	**2.7**	**1.2**	**2.5**	**1.4**	**1.3**	**.259**
To what extent did you feel surprised by the course of events in delivery?	**3.1**	**1.0**	**2.7**	**1.4**	**3.6**	**.061**
To what extent did you feel your life was in danger?	**1.3**	**1.5**	**1.4**	**1.5**	**0.2**	**.639**
To what extent did you feel your baby's life was in danger?	**1.8**	**1.4**	**1.8**	**1.4**	**0.1**	**.739**
To what extent did you feel that following this delivery you would like your next delivery to be by cesarean section?	**1.7**	**1.5**	**1.3**	**1.6**	**3.3**	**.070**
To what extent did you feel that following this delivery you don’t want to have any more children?	**1.0**	**1.2**	**.9**	**1.3**	**0.4**	**.507**

*Note*: Ns differed along questions due to missing data.

^+^The scoring was reversed: a higher score denotes a more negative birth experience- less control.

## Discussion

We report that women who had a planned delivery (VD or elective CS) perceived their childbirth experience more positively than did those who delivered by unplanned interventions (emergency CS or VE). Conversely, despite the intrusive nature and medical risks of emergency CS, compared to VE, the perceived childbirth experience was similar between women who delivered by these two modes of unplanned intervention; and similar also among women who delivered as planned (vaginally or by elective CS). These findings concur with studies that found emergency CS to be a strong predictor of negative birth experience [7, 14–16]; and studies that showed the birth experience to be equally positive in elective CS and in VD, and more positive than the experience in emergency CS and VE [17, 18].

Previous studies indicated that lack of control, lack of involvement in decision-making, and expectations not being met are some of the factors associated with negative birth experience [10, 11, 19, 20]. These characteristics may be associated with unplanned emergency interventions such as VE and emergency CS. The current findings are consistent with the notion that events are experienced as stressful when they are sudden, dangerous, and overwhelming [21].

In the present study, women who underwent emergency interventions due to delay of birth (arrest of labor) perceived their birth experience more negatively than did those who underwent interventions due to risk for the mother or fetus, regardless of the nature of the intervention (VE or emergency CS). Analysis of the specific items of the SCE questionnaire show that women who underwent arrested labor felt that the delivery was more difficult and expressed more helplessness and less control, compared to those who underwent interventions due to maternal or fetal risks. These findings seem to reflect the ambiguity around the decision to intervene when the indication is due to arrest of labor. In such circumstances, women may feel that the determination of intervention is due to their own actions. This possible sense of helplessness, lack of control, and difficulty could elicit negative feelings. In contrast, when interventions are determined by medical teams due to a clear risk to the mother or fetus, women may be less likely to interpret the circumstances of the intervention as related to their own actions, and may thus perceive the birth experience less negatively. Such possible explanations should be further explored in future research.

The clinical significance of this study is related to the fact that a negative birth experience may have repercussions in a woman's psychosocial life and well-being. Negative birth experience has been linked to postpartum depression and post-trauma [4, 6], fear of subsequent birth, and a wish for future elective CS [22, 23]. In some cases, negative birth experience may increase the likelihood of not wanting more children [24]. This study supports our feeling that more attention should be given to women's perceptions of their childbirth experience, with follow-up for women with negative perceptions. Particular attention to personal well-being should be given to women who undergo emergency procedures, and especially when they are due to arrest disorders and to the emotions and perceptions that might lead to a negative birth experience, such as lack of information and support, lack of shared decision-making, loss of control and feeling of failure.

This study was limited by its correlational nature, which prevented us to determine cause-and-effect relationship and a possible selection bias owing to the use of a convenience sample. Another limitation concerns the stress-oriented negative nature of the childbirth questionnaire used in this study. Future studies should employ questionnaires targeted at both the positive as well the negative nature of childbirth experience. Furthermore, our findings may not be generalizable to other populations.

In conclusion, we report that unexpected mode of delivery (VE or emergency CS) results in a more negative birth experience than a planned mode of delivery. Moreover, women who undergo delivery procedures that result from arrest indications perceive a more negative childbirth experience than those for whom there is an immediate risk to the mother or fetus, regardless of the particular nature of the intervention. The overall difference in childbirth experience reported here stemmed from the women’s perception of a more difficult delivery and their sense of helplessness and less control. Further studies regarding the psychological factors associated with the different indications for delivery, such as the degree of control and fear, are needed to fully understand the birth experience and the possible contribution to post-partum wellbeing.
